# *catena*-Poly[[bis­(2-meth­oxy­aniline-κ*N*)cadmium]-di-μ-thio­cyanato-κ^2^ *N*:*S*;κ^2^ *S*:*N*]

**DOI:** 10.1107/S1600536813010738

**Published:** 2013-04-27

**Authors:** Rakia Chemli, Slaheddine Kamoun, Thierry Roisnel

**Affiliations:** a Laboratoire de Génie des Matériaux et Environnement, École Nationale d’Ingénieurs de Sfax, BP 1173, Sfax, Tunisia; b Centre de Diffractométrie X, UMR 6226 CNRS Unitée Sciences, Chimiques de Rennes, Université de Rennes I, 263 Avenue du Général Leclerc, 35042 Rennes, France

## Abstract

The structure of the title compound, [Cd(NCS)_2_(C_7_H_9_NO)_2_]_*n*_, consists of cadmium–thio­cyanate layers parallel to the *ab* plane. Pairs of Cd^II^ ions are bridged by two end-to-end inversely bridging μ-NCS-*N*:*S* thio­cyanate groups, forming a two-dimensional network with the remaining two *trans* positions of the octa­hedrally coordinated Cd^II^ ions occupied by the N atoms of two neutral 2-meth­oxy­aniline ligands. The crystal structure is stabilized by intra­layer N—H⋯S hydrogen bonds.

## Related literature

For related structures, see: Wöhlert *et al.* (2012 ▶, 2013 ▶); Bai *et al.* (2011 ▶); Yang *et al.* (2001 ▶). For HSCN synthesis, see: Bartlett *et al.* (1969 ▶). For the effects of substituents on the inter­nal angles of the benzene ring, see: Domenicano & Murray-Rust (1979 ▶). For non-linear optical and luminescence properties of related compounds, see: Chen *et al.* (2000 ▶); Bai *et al.* (2011 ▶). For electric and dielectric properties of related compounds, see: Karoui *et al.* (2013 ▶).
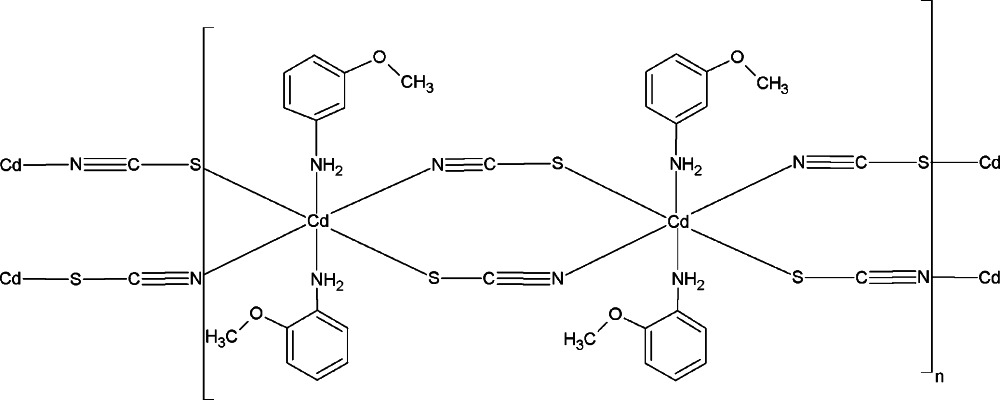


## Experimental

### 

#### Crystal data

[Cd(NCS)_2_(C_7_H_9_NO)_2_]
*M* *_r_* = 474.89
Orthorhombic, 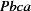

*a* = 6.6860 (2) Å
*b* = 23.3658 (7) Å
*c* = 24.3281 (8) Å
*V* = 3800.6 (2) Å^3^
*Z* = 8
Mo *K*α radiation
μ = 1.39 mm^−1^
*T* = 150 K
0.33 × 0.18 × 0.11 mm

#### Data collection

Bruker APEXII diffractometer
Absorption correction: multi-scan (*SADABS*; Bruker, 2011 ▶) *T*_min_ = 0.741, *T*_max_ = 0.859
16897 measured reflections
4210 independent reflections
3262 reflections with *I* > 2σ(*I*)
*R*_int_ = 0.031

#### Refinement

*R*[*F* ^2^ > 2σ(*F* ^2^)] = 0.025
*wR*(*F* ^2^) = 0.055
*S* = 1.02
4210 reflections
228 parameters
H-atom parameters constrained
Δρ_max_ = 0.35 e Å^−3^
Δρ_min_ = −0.38 e Å^−3^

### 

Data collection: *APEX2* (Bruker, 2011 ▶); cell refinement: *SAINT* (Bruker, 2011 ▶); data reduction: *SAINT*; program(s) used to solve structure: *SHELXS97* (Sheldrick, 2008 ▶); program(s) used to refine structure: *SHELXL97* (Sheldrick, 2008 ▶); molecular graphics: *DIAMOND* (Brandenburg & Berndt, 2001 ▶) and *Mercury* (Macrae *et al.*, 2008 ▶); software used to prepare material for publication: *WinGX* (Farrugia, 2012 ▶) and *publCIF* (Westrip, 2010 ▶).

## Supplementary Material

Crystal structure: contains datablock(s) I, global. DOI: 10.1107/S1600536813010738/rz5058sup1.cif

Structure factors: contains datablock(s) I. DOI: 10.1107/S1600536813010738/rz5058Isup2.hkl

Supplementary material file. DOI: 10.1107/S1600536813010738/rz5058Isup3.cdx

Additional supplementary materials:  crystallographic information; 3D view; checkCIF report

## Figures and Tables

**Table 1:** Selected bond lengths (Å)

Cd1—N2	2.2724 (19)
Cd1—N1	2.3142 (18)
Cd1—N11	2.3653 (17)
Cd1—N21	2.3718 (17)
Cd1—S1^i^	2.7306 (6)
Cd1—S2^ii^	2.7449 (6)

Symmetry codes: (i) 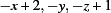; (ii) 
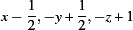
.

**Table 2:** Hydrogen-bond geometry (Å, °)

*D*—H⋯*A*	*D*—H	H⋯*A*	*D*⋯*A*	*D*—H⋯*A*
N11—H11*A*⋯S2^iii^	0.92	2.50	3.4182 (17)	174
N21—H21*B*⋯S1^iv^	0.92	2.66	3.5637 (18)	169

Symmetry codes: (iii) 
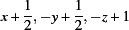
; (iv) 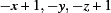.

**Fig. 1.**
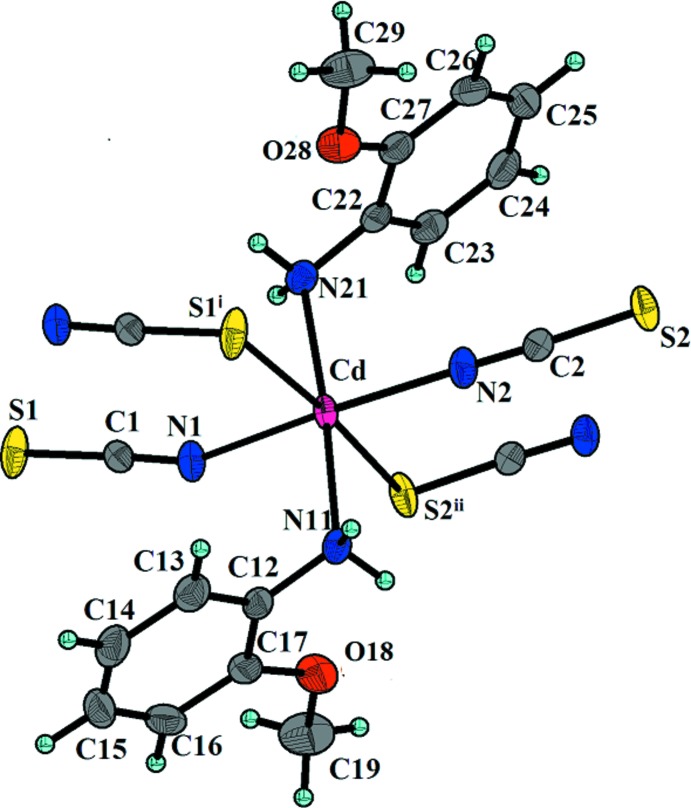
Crystal structure of the title compound, showing the coordination around the Cd2+ cation with displacement ellipsoids drawn at the 50% probability level.

**Fig. 2.**
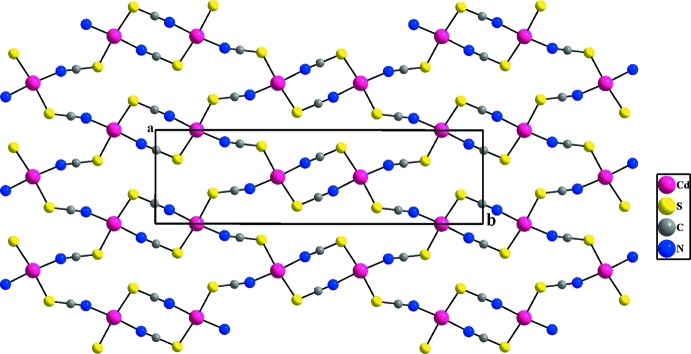
View at z=0 of the two-dimensional layer structure of the inorganic coordination polymer parallel to the ab plane. The organic ligands are omitted for clarity.

## supplementary crystallographic information

### Comment

The crystal engineering of inorganic-organic hybrid coordination polymers is currently one of the most active fields in coordination chemistry, supramolecular and materials chemistry. These compounds attract significant attention for their architectures and topologies (Yang *et al.*, 2001). Hybrid inorganic-organic thiocyanate materials exhibit interesting physical properties such electrical conductivity and dielectric relaxation process (Karoui *et al.*, 2013) and may have potential applications in non-linear optics and luminescence (Chen *et al.*, 2000; Bai *et al.*, 2011). Herein we report the structure of a new polymeric hybrid title compound

As shown in Fig. 1, each cadmium atom, which sits on general position, is coordinated by two *trans* N-bonded and two *trans* S-bonded thiocyanato anions. Two *trans*-coordinated 2-methoxy aniline ligands complete the octahedral coordination geometry around cadmium. The crystal structure of the title compound consists of both simply and doubly µ-1,3-SCN–bridged two dimensional networks parallel to the *ab* plane with terminal neutral 2-methoxyaniline ligands (Fig. 2). In the CdN_4_S_2_ core, the Cd—N and Cd—S bonds span the range 2.2724 (19)–2.3718 (17) Å and 2.7306 (6)–2.7449 (6) Å, respectively (Table 1). These values are in good agreement with those observed in other similar complexes (Wöhlert *et al.*, 2012, 2013; Yang *et al.*, 2001). The bond angles involving the cadmium(II) cation range from 84.29 (6) to 95.50 (7)° and from 174.91 (2) to 175.76 (6)°. The double-bridging role of S1/C1/N1 anions gives rise to eight-membered Cd_2_(SCN)_2_ rings in a chair conformation because of the almost linear SCN groups (S1–C1–N1 angle = 178.76 (21)°). The distance between adjacent Cd atoms in the Cd_2_(SCN)_2_ rings is 5.9127 (4) Å. Again, these rings built up a 2-D sheet through their corner-sharing action at the two cadmium atoms *via* the linear S2/C2/N2 anions (S1–C1–N1 angle = 177.96 (21)°) and give rise to thirty-membered [Cd_6_(µ-SCN-*N*,*S*)_8_] macrocycles as subunits, as shown in Fig. 2. The bond angles in the phenyl groups deviate significantly from the idealized value of 120° due to the effect of the substituent. In fact, it was established that the angular deformations of phenyl groups can be described as a sum of the effects of the different substituents (Domenicano & Murray-Rust, 1979). The phenyl rings of both independent 2-methoxyaniline ligands are planar with the greatest deviation from the six-atoms least-square plane of 0.0029 Å and 0.0073 Å. They are well ordered with C–C–C angles in agreement with the expected *sp*^2^ hybridation. The π-π interactions between phenyl rings may be neglected (>4 Å); in fact the shortest distances between the centroids of the rings are: Cg1 ··· Cg1^i^ = 5.6130 (14) Å; Cg1 ··· Cg2^ii^ = 5.5949 (14) Å (Cg1 and Cg2 are the centroids of the C12–C17 and C22–C27 rings, respectively; symmetry codes: (i) -0.5+x, +y, 1.5-z; 2.-x, -y, 1.-z). The major contributions to the cohesion and the stability of the polymeric structure is the presence of intralayer N—H···S hydrogen bonds which include two relatively long contacts, with H···S and N..S distances ranging from 2.50 to 2.66 Å and 3.4182 (17) Å to 3.5637 (18) Å, respectively (Table 2).

### Experimental

To 25 ml of an aqueous solution of thiocyanic acid (0.8 M) prepared using the published procedure (Bartlett *et al.*, 1969) an appropriate amount of cadmium carbonate (1.664 g, 10 mmol) was added and refluxed for 3 h. After cooling, 25 ml of methanol and 2.3 ml of a solution of 2-methoxyaniline (8.64 M) was added. The resulting solution was heated under reflux for 1 h and left to stand at ambient temperature. Well shaped colourless crystals were obtained after 2 h on slow evaporation of the solvent. They were washed with diethyl ether and dried over P_2_O_5_.

### Refinement

All H atoms were positioned geometrically and allowed to ride on their parent atoms, with C—H = 0.95-0.98 Å, N—H = 0.92 Å, and with *U*_iso_(H) = 1.2*U*_eq_(C, N).

### Crystal data

**Table:**

[Cd(NCS)_2_(C_7_H_9_NO)_2_]	*F*(000) = 1904
*M**_r_* = 474.89	*D*_x_ = 1.660 Mg m^−^^3^*D*_m_ = 1.593 Mg m^−^^3^*D*_m_ measured by flotation
Orthorhombic, *P**b**c**a*	Mo *K*α radiation, λ = 0.71073 Å
Hall symbol: -P 2ac 2ab	Cell parameters from 5155 reflections
*a* = 6.6860 (2) Å	θ = 3.1–27.4°
*b* = 23.3658 (7) Å	µ = 1.39 mm^−^^1^
*c* = 24.3281 (8) Å	*T* = 150 K
*V* = 3800.6 (2) Å^3^	Prism, colourless
*Z* = 8	0.33 × 0.18 × 0.11 mm

### Data collection

**Table:**

Bruker APEXII diffractometer	3262 reflections with *I* > 2σ(*I*)
Radiation source: fine-focus sealed tube	*R*_int_ = 0.031
Graphite monochromator	θ_max_ = 27.5°, θ_min_ = 3.1°
CCD rotation images, thin slices scans	*h* = −8→8
Absorption correction: multi-scan (*SADABS*; Bruker, 2011)	*k* = −29→30
*T*_min_ = 0.741, *T*_max_ = 0.859	*l* = −31→20
16897 measured reflections	2 standard reflections every 120 min
4210 independent reflections	

### Refinement

**Table:**

Refinement on *F*^2^	Primary atom site location: structure-invariant direct methods
Least-squares matrix: full	Secondary atom site location: difference Fourier map
*R*[*F*^2^ > 2σ(*F*^2^)] = 0.025	Hydrogen site location: inferred from neighbouring sites
*wR*(*F*^2^) = 0.055	H-atom parameters constrained
*S* = 1.02	*w* = 1/[σ^2^(*F*_o_^2^) + (0.0184*P*)^2^ + 1.4548*P*] where *P* = (*F*_o_^2^ + 2*F*_c_^2^)/3
4210 reflections	(Δ/σ)_max_ = 0.004
228 parameters	Δρ_max_ = 0.35 e Å^−^^3^
0 restraints	Δρ_min_ = −0.38 e Å^−^^3^

### Special details

**Table:**

Geometry. All e.s.d.'s (except the e.s.d. in the dihedral angle between two l.s. planes) are estimated using the full covariance matrix. The cell e.s.d.'s are taken into account individually in the estimation of e.s.d.'s in distances, angles and torsion angles; correlations between e.s.d.'s in cell parameters are only used when they are defined by crystal symmetry. An approximate (isotropic) treatment of cell e.s.d.'s is used for estimating e.s.d.'s involving l.s. planes.
Refinement. Refinement of *F*^2^ against ALL reflections. The weighted *R*-factor *wR* and goodness of fit *S* are based on *F*^2^, conventional *R*-factors *R* are based on *F*, with *F* set to zero for negative *F*^2^. The threshold expression of *F*^2^ > σ(*F*^2^) is used only for calculating *R*-factors(gt) *etc*. and is not relevant to the choice of reflections for refinement. *R*-factors based on *F*^2^ are statistically about twice as large as those based on *F*, and *R*- factors based on ALL data will be even larger.

### Fractional atomic coordinates and isotropic or equivalent isotropic displacement parameters (Å^2^)

**Table:**

	*x*	*y*	*z*	*U*_iso_*/*U*_eq_	
Cd1	0.99993 (2)	0.126200 (7)	0.508711 (7)	0.02024 (6)	
S1	0.68873 (8)	−0.06757 (2)	0.53717 (3)	0.03279 (16)	
C1	0.7845 (3)	−0.00256 (9)	0.53832 (8)	0.0191 (5)	
N1	0.8515 (3)	0.04293 (8)	0.54015 (8)	0.0253 (4)	
S2	1.17331 (8)	0.32408 (2)	0.44237 (3)	0.02739 (14)	
N2	1.1259 (3)	0.21155 (8)	0.48030 (8)	0.0266 (4)	
C2	1.1461 (3)	0.25807 (9)	0.46565 (9)	0.0193 (5)	
N11	1.1959 (2)	0.13518 (7)	0.58919 (7)	0.0213 (4)	
H11A	1.3247	0.1440	0.5789	0.026*	
H11B	1.1478	0.1657	0.6092	0.026*	
C12	1.2028 (3)	0.08631 (9)	0.62450 (9)	0.0220 (5)	
C13	1.3485 (3)	0.04507 (10)	0.61900 (10)	0.0312 (6)	
H13	1.4520	0.0498	0.5926	0.037*	
C14	1.3446 (4)	−0.00363 (11)	0.65207 (11)	0.0412 (7)	
H14	1.4466	−0.0317	0.6488	0.049*	
C15	1.1926 (5)	−0.01091 (11)	0.68948 (11)	0.0441 (7)	
H15	1.1889	−0.0445	0.7115	0.053*	
C16	1.0444 (4)	0.03030 (12)	0.69545 (9)	0.0362 (6)	
H16	0.9394	0.0249	0.7213	0.043*	
C17	1.0510 (3)	0.07931 (10)	0.66349 (9)	0.0258 (5)	
O18	0.9195 (2)	0.12405 (7)	0.66597 (7)	0.0344 (4)	
C19	0.7522 (4)	0.11868 (13)	0.70215 (11)	0.0478 (8)	
H19A	0.6797	0.0833	0.6938	0.072*	
H19B	0.6628	0.1515	0.6972	0.072*	
H19C	0.7994	0.1176	0.7403	0.072*	
N21	0.8084 (3)	0.10925 (8)	0.42829 (7)	0.0240 (4)	
H21A	0.8731	0.0813	0.4085	0.029*	
H21B	0.6879	0.0941	0.4393	0.029*	
C22	0.7653 (3)	0.15518 (10)	0.39121 (9)	0.0230 (5)	
C23	0.5968 (3)	0.18854 (10)	0.39643 (9)	0.0281 (5)	
H23	0.5026	0.1805	0.4246	0.034*	
C24	0.5637 (4)	0.23401 (11)	0.36050 (10)	0.0344 (6)	
H24	0.4466	0.2568	0.3640	0.041*	
C25	0.7010 (4)	0.24592 (11)	0.31981 (10)	0.0380 (6)	
H25	0.6786	0.2771	0.2955	0.046*	
C26	0.8727 (4)	0.21251 (11)	0.31418 (9)	0.0346 (6)	
H26	0.9674	0.2209	0.2862	0.042*	
C27	0.9044 (3)	0.16704 (10)	0.34953 (9)	0.0269 (5)	
O28	1.0642 (2)	0.13068 (7)	0.34823 (7)	0.0348 (4)	
C29	1.1988 (4)	0.13507 (13)	0.30289 (10)	0.0446 (7)	
H29A	1.1241	0.1312	0.2684	0.067*	
H29B	1.2992	0.1046	0.3053	0.067*	
H29C	1.2655	0.1724	0.3038	0.067*	

### Atomic displacement parameters (Å^2^)

**Table:**

	*U*^11^	*U*^22^	*U*^33^	*U*^12^	*U*^13^	*U*^23^
Cd1	0.02022 (9)	0.01308 (9)	0.02743 (10)	−0.00211 (6)	0.00012 (7)	0.00258 (7)
S1	0.0237 (3)	0.0153 (3)	0.0594 (4)	−0.0043 (2)	0.0168 (3)	−0.0044 (3)
C1	0.0164 (9)	0.0193 (12)	0.0217 (11)	0.0047 (9)	0.0036 (9)	0.0024 (10)
N1	0.0238 (9)	0.0161 (10)	0.0361 (11)	0.0001 (8)	0.0062 (9)	0.0016 (9)
S2	0.0211 (3)	0.0175 (3)	0.0435 (3)	−0.0045 (2)	−0.0093 (3)	0.0096 (3)
N2	0.0265 (10)	0.0169 (10)	0.0364 (11)	−0.0047 (8)	0.0035 (9)	0.0010 (9)
C2	0.0143 (9)	0.0202 (12)	0.0235 (11)	−0.0014 (9)	−0.0001 (9)	−0.0028 (10)
N11	0.0192 (8)	0.0177 (10)	0.0272 (10)	−0.0018 (8)	0.0048 (8)	−0.0019 (8)
C12	0.0241 (10)	0.0196 (11)	0.0224 (11)	−0.0026 (9)	−0.0044 (9)	−0.0025 (10)
C13	0.0339 (12)	0.0283 (13)	0.0314 (13)	0.0025 (11)	−0.0080 (11)	−0.0051 (12)
C14	0.0588 (17)	0.0254 (14)	0.0392 (15)	0.0107 (13)	−0.0238 (14)	−0.0068 (13)
C15	0.077 (2)	0.0220 (14)	0.0334 (15)	−0.0083 (14)	−0.0267 (15)	0.0048 (12)
C16	0.0501 (15)	0.0380 (16)	0.0205 (12)	−0.0184 (13)	−0.0058 (11)	0.0031 (12)
C17	0.0309 (11)	0.0254 (13)	0.0211 (11)	−0.0053 (10)	−0.0024 (10)	−0.0010 (11)
O18	0.0304 (8)	0.0399 (11)	0.0330 (9)	0.0006 (8)	0.0131 (8)	0.0038 (8)
C19	0.0336 (14)	0.070 (2)	0.0400 (16)	−0.0100 (14)	0.0149 (12)	−0.0017 (16)
N21	0.0248 (9)	0.0219 (10)	0.0253 (10)	−0.0043 (8)	0.0037 (8)	−0.0030 (9)
C22	0.0289 (11)	0.0201 (12)	0.0201 (11)	−0.0065 (10)	−0.0032 (9)	−0.0052 (10)
C23	0.0294 (12)	0.0292 (13)	0.0259 (12)	−0.0053 (11)	−0.0023 (10)	−0.0073 (11)
C24	0.0422 (13)	0.0243 (13)	0.0366 (14)	0.0016 (12)	−0.0112 (12)	−0.0102 (12)
C25	0.0625 (17)	0.0245 (14)	0.0270 (13)	−0.0068 (13)	−0.0123 (13)	0.0007 (12)
C26	0.0503 (15)	0.0342 (15)	0.0194 (12)	−0.0115 (13)	0.0036 (11)	−0.0036 (12)
C27	0.0312 (12)	0.0263 (13)	0.0231 (12)	−0.0055 (11)	−0.0005 (10)	−0.0060 (11)
O28	0.0319 (8)	0.0434 (11)	0.0290 (9)	0.0008 (8)	0.0089 (7)	−0.0014 (8)
C29	0.0358 (14)	0.066 (2)	0.0321 (14)	−0.0049 (14)	0.0089 (12)	−0.0082 (14)

### Geometric parameters (Å, º)

**Table:**

Cd1—N2	2.2724 (19)	C16—H16	0.9500
Cd1—N1	2.3142 (18)	C17—O18	1.367 (3)
Cd1—N11	2.3653 (17)	O18—C19	1.429 (3)
Cd1—N21	2.3718 (17)	C19—H19A	0.9800
Cd1—S1^i^	2.7306 (6)	C19—H19B	0.9800
Cd1—S2^ii^	2.7449 (6)	C19—H19C	0.9800
S1—C1	1.649 (2)	N21—C22	1.431 (3)
S1—Cd1^i^	2.7306 (6)	N21—H21A	0.9200
C1—N1	1.154 (3)	N21—H21B	0.9200
S2—C2	1.653 (2)	C22—C23	1.376 (3)
S2—Cd1^iii^	2.7449 (5)	C22—C27	1.403 (3)
N2—C2	1.152 (3)	C23—C24	1.394 (3)
N11—C12	1.430 (3)	C23—H23	0.9500
N11—H11A	0.9200	C24—C25	1.379 (3)
N11—H11B	0.9200	C24—H24	0.9500
C12—C13	1.377 (3)	C25—C26	1.395 (3)
C12—C17	1.399 (3)	C25—H25	0.9500
C13—C14	1.394 (3)	C26—C27	1.383 (3)
C13—H13	0.9500	C26—H26	0.9500
C14—C15	1.375 (4)	C27—O28	1.365 (3)
C14—H14	0.9500	O28—C29	1.427 (3)
C15—C16	1.389 (4)	C29—H29A	0.9800
C15—H15	0.9500	C29—H29B	0.9800
C16—C17	1.385 (3)	C29—H29C	0.9800
			
N2—Cd1—N1	175.76 (6)	O18—C17—C16	125.9 (2)
N2—Cd1—N11	88.20 (6)	O18—C17—C12	114.0 (2)
N1—Cd1—N11	92.22 (6)	C16—C17—C12	120.0 (2)
N2—Cd1—N21	95.50 (7)	C17—O18—C19	117.6 (2)
N1—Cd1—N21	84.29 (6)	O18—C19—H19A	109.5
N11—Cd1—N21	175.44 (6)	O18—C19—H19B	109.5
N2—Cd1—S1^i^	91.92 (5)	H19A—C19—H19B	109.5
N1—Cd1—S1^i^	92.31 (4)	O18—C19—H19C	109.5
N11—Cd1—S1^i^	87.74 (4)	H19A—C19—H19C	109.5
N21—Cd1—S1^i^	89.47 (5)	H19B—C19—H19C	109.5
N2—Cd1—S2^ii^	93.16 (5)	C22—N21—Cd1	120.21 (13)
N1—Cd1—S2^ii^	82.60 (4)	C22—N21—H21A	107.3
N11—Cd1—S2^ii^	92.54 (4)	Cd1—N21—H21A	107.3
N21—Cd1—S2^ii^	89.92 (5)	C22—N21—H21B	107.3
S1^i^—Cd1—S2^ii^	174.914 (18)	Cd1—N21—H21B	107.3
C1—S1—Cd1^i^	99.97 (7)	H21A—N21—H21B	106.9
N1—C1—S1	178.8 (2)	C23—C22—C27	119.8 (2)
C1—N1—Cd1	158.10 (18)	C23—C22—N21	122.1 (2)
C2—S2—Cd1^iii^	109.52 (7)	C27—C22—N21	118.0 (2)
C2—N2—Cd1	164.95 (17)	C22—C23—C24	120.3 (2)
N2—C2—S2	178.0 (2)	C22—C23—H23	119.9
C12—N11—Cd1	116.38 (13)	C24—C23—H23	119.9
C12—N11—H11A	108.2	C25—C24—C23	119.9 (2)
Cd1—N11—H11A	108.2	C25—C24—H24	120.0
C12—N11—H11B	108.2	C23—C24—H24	120.0
Cd1—N11—H11B	108.2	C24—C25—C26	120.4 (2)
H11A—N11—H11B	107.3	C24—C25—H25	119.8
C13—C12—C17	119.8 (2)	C26—C25—H25	119.8
C13—C12—N11	121.6 (2)	C27—C26—C25	119.7 (2)
C17—C12—N11	118.52 (19)	C27—C26—H26	120.2
C12—C13—C14	120.1 (2)	C25—C26—H26	120.2
C12—C13—H13	119.9	O28—C27—C26	125.7 (2)
C14—C13—H13	119.9	O28—C27—C22	114.4 (2)
C15—C14—C13	119.8 (2)	C26—C27—C22	120.0 (2)
C15—C14—H14	120.1	C27—O28—C29	117.82 (19)
C13—C14—H14	120.1	O28—C29—H29A	109.5
C14—C15—C16	120.7 (2)	O28—C29—H29B	109.5
C14—C15—H15	119.6	H29A—C29—H29B	109.5
C16—C15—H15	119.6	O28—C29—H29C	109.5
C17—C16—C15	119.4 (2)	H29A—C29—H29C	109.5
C17—C16—H16	120.3	H29B—C29—H29C	109.5
C15—C16—H16	120.3		
			
N11—Cd1—N1—C1	−141.7 (4)	C13—C12—C17—C16	−1.7 (3)
N21—Cd1—N1—C1	35.3 (4)	N11—C12—C17—C16	175.03 (19)
S1^i^—Cd1—N1—C1	−53.9 (4)	C16—C17—O18—C19	−4.3 (3)
S2^ii^—Cd1—N1—C1	126.0 (4)	C12—C17—O18—C19	176.0 (2)
N11—Cd1—N2—C2	−122.1 (7)	N2—Cd1—N21—C22	−16.79 (16)
N21—Cd1—N2—C2	60.6 (7)	N1—Cd1—N21—C22	158.96 (16)
S1^i^—Cd1—N2—C2	150.2 (7)	S1^i^—Cd1—N21—C22	−108.66 (15)
S2^ii^—Cd1—N2—C2	−29.6 (7)	S2^ii^—Cd1—N21—C22	76.39 (15)
N2—Cd1—N11—C12	−171.56 (15)	Cd1—N21—C22—C23	−90.3 (2)
N1—Cd1—N11—C12	12.66 (14)	Cd1—N21—C22—C27	87.6 (2)
S1^i^—Cd1—N11—C12	−79.56 (14)	C27—C22—C23—C24	0.0 (3)
S2^ii^—Cd1—N11—C12	95.35 (14)	N21—C22—C23—C24	177.95 (19)
Cd1—N11—C12—C13	91.3 (2)	C22—C23—C24—C25	−0.5 (3)
Cd1—N11—C12—C17	−85.4 (2)	C23—C24—C25—C26	0.4 (4)
C17—C12—C13—C14	0.2 (3)	C24—C25—C26—C27	0.3 (4)
N11—C12—C13—C14	−176.4 (2)	C25—C26—C27—O28	179.3 (2)
C12—C13—C14—C15	1.3 (4)	C25—C26—C27—C22	−0.8 (3)
C13—C14—C15—C16	−1.2 (4)	C23—C22—C27—O28	−179.42 (19)
C14—C15—C16—C17	−0.3 (4)	N21—C22—C27—O28	2.6 (3)
C15—C16—C17—O18	−178.0 (2)	C23—C22—C27—C26	0.6 (3)
C15—C16—C17—C12	1.7 (3)	N21—C22—C27—C26	−177.39 (19)
C13—C12—C17—O18	178.09 (19)	C26—C27—O28—C29	−8.1 (3)
N11—C12—C17—O18	−5.2 (3)	C22—C27—O28—C29	172.00 (19)

Symmetry codes: (i) −*x*+2, −*y*, −*z*+1; (ii) *x*−1/2, −*y*+1/2, −*z*+1; (iii) *x*+1/2, −*y*+1/2, −*z*+1.

### Hydrogen-bond geometry (Å, º)

**Table:**

*D*—H···*A*	*D*—H	H···*A*	*D*···*A*	*D*—H···*A*
N11—H11*A*···S2^iii^	0.92	2.50	3.4182 (17)	174
N21—H21*B*···S1^iv^	0.92	2.66	3.5637 (18)	169
N11—H11*B*···O18	0.92	2.28	2.641 (2)	103
N21—H21*A*···O28	0.92	2.26	2.640 (2)	104

Symmetry codes: (iii) *x*+1/2, −*y*+1/2, −*z*+1; (iv) −*x*+1, −*y*, −*z*+1.
